# Acetaminophen (Paracetamol) Exposure During Pregnancy and Pubertal Development in Boys and Girls From a Nationwide Puberty Cohort

**DOI:** 10.1093/aje/kwy193

**Published:** 2018-09-07

**Authors:** Andreas Ernst, Nis Brix, Lea L B Lauridsen, Jørn Olsen, Erik T Parner, Zeyan Liew, Lars H Olsen, Cecilia H Ramlau-Hansen

**Affiliations:** 1 Section for Epidemiology, Department of Public Health, Aarhus University, Aarhus, Denmark; 2 Department of Epidemiology, Fielding School of Public Health, University of California Los Angeles, Los Angeles, California; 3 Department of Clinical Epidemiology, Aarhus University Hospital, Aarhus, Denmark; 4 Section for Biostatistics, Department of Public Health, Aarhus University, Aarhus, Denmark; 5 Section for Paediatric Urology, Department of Urology, Aarhus University Hospital, Aarhus, Denmark

**Keywords:** endocrine disrupters, prenatal exposure delayed effects, puberty, sex characteristics, Tanner stages

## Abstract

This study explored the association between exposure to acetaminophen during pregnancy and pubertal development using data from 15,822 boys and girls in the longitudinal Puberty Cohort, nested within the Danish National Birth Cohort. Use of acetaminophen was reported 3 times during pregnancy and 6 months postpartum. In total, 54% of mothers indicated use at least once during pregnancy. Between 2012 and 2017, sons and daughters provided information on a wide range of pubertal milestones—including Tanner stages, axillary hair growth, and age at menarche or voice break and first ejaculation—every 6 months from 11 years of age until full sexual maturation. Data were analyzed using a regression model for interval-censored data, providing adjusted mean monthly differences in age at attaining the pubertal milestones according to intrauterine cumulative (weeks) and trimester-specific acetaminophen exposure. Our results suggested a tendency towards slightly earlier attainment of almost all studied markers of female pubertal development with increasing number of weeks of exposure (i.e., about 1.5–3 months earlier age at pubic hair, axillary hair, and acne development comparing unexposed with those prenatally exposed for more than 12 weeks). Male pubertal development had no strong association with acetaminophen exposure.

Use of over-the-counter analgesics, especially acetaminophen, has been steadily increasing in Western countries (1). Acetaminophen, also known as paracetamol, is now being used at least once by more than 50% of pregnant women in some countries (2–4). It is considered safe by the general public and remains a first choice for treatment for fever and pain during pregnancy (5).

Acetaminophen is, however, capable of crossing the placental barrier (6, 7) and might therefore interfere with organ development affecting health in the offspring, including reproductive health (8). Studies in rodents suggest that administration of acetaminophen equivalent to human therapeutic doses exerts antiandrogenic disruption, inhibiting early masculinization of male rats (9–12). On the other hand, an in vitro study of human fetal testicular tissue found no association between short-term exposure to acetaminophen and testosterone production (13). A recent study in rodents linked intrauterine exposure to acetaminophen with alterations in fetal germ cell development and differentiation, reducing later reproductive capacity in female—but not in male—offspring (14). Cohort studies on the associations between prenatal exposure to acetaminophen and anogenital distance support evidence from animal studies of antiandrogenic disruption in males but do not demonstrate a similar association in females (15, 16). In parallel, studies on male genital malformations show no associations with hypospadias and inconclusive results concerning cryptorchidism (9, 17–22). It remains largely unknown to what extent a potential endocrine programming disruption of intrauterine exposure to acetaminophen might affect long-term reproductive health, such as age at puberty timing. To our knowledge, this is the first study on puberty timing, and we hypothesize that intrauterine exposure to acetaminophen alters the timing of markers of male and female pubertal development.

## METHODS

### Study population

Our study used data from the large nationwide Puberty Cohort that is nested within the Danish National Birth Cohort (DNBC) (23). The pregnant women in DNBC were recruited from 1996 to 2002. Half of all general practitioners in Denmark participated in the recruitment process, and about 60% of the pregnant women accepted the invitation handed out at their first antenatal visit. A self-administrated enrollment form and 4 computer-assisted telephone interviews on lifestyle during pregnancy and health of their children were carried out at approximately gestational weeks 17 and 32 as well as 6 and 18 months after birth.

**Figure 1.**
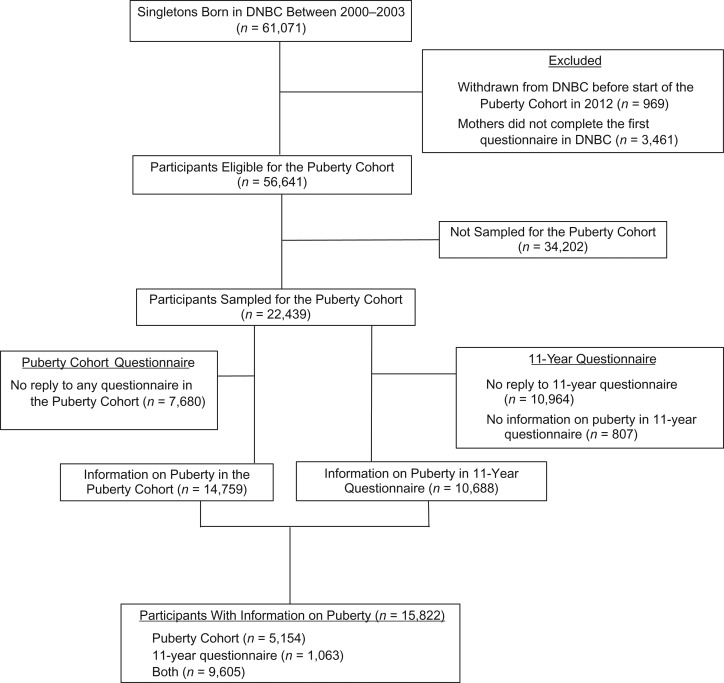
Flow diagram of participants in the Puberty Cohort nested within the Danish National Birth Cohort (DNBC) (*n* = 15,822), Denmark, 2000–2017.

A total of 56,641 live-born singletons born to mothers in DNBC from 2000 to 2003 were eligible for participation in the Puberty Cohort. The mothers had responded to the first questionnaire in DNBC and not withdrawn their consent before 2012 (Figure 1). An oversampling strategy was used to create the Puberty Cohort, aiming to increase statistical efficiency by ensuring adequate samples sizes. We oversampled participants from subgroups of 12 pre- and perinatal exposures (27 sampling frames) considered to be of relevance for timing of puberty and paired these with a randomly selected reference group of 8,000 children (1 sampling frame), making up the Puberty Cohort of 22,439 children. The relevant pre- and perinatal exposures included maternal gestational use of acetaminophen.

At 11.5 years of age, participating children were invited to provide information on their current stage of puberty every 6 months until they reached full sexual maturation or turned 18 years of age, whichever came first. Full sexual maturation was defined as self-reported Tanner stage 5 for both pubic hair growth and breast or genital development (24, 25).

To complement the information on puberty timing in the Puberty Cohort, we added data from an 11-year follow-up of all children in the DNBC. Altogether 10,668 children, subsequently sampled for the Puberty Cohort, responded to a comprehensive questionnaire that also included similar questions on pubertal development. In total, when data from the 11-year questionnaire was added, information on pubertal development of 15,822 children from the Puberty Cohort was available.

### Exposure assessment

Information on use of acetaminophen was retrieved through the maternal enrollment form and 3 of the computer-assisted telephone interviews, covering use from 4 weeks before pregnancy until delivery. The enrollment form included questions on use of painkillers and specifications on timing of use until the 14th week of pregnancy. In the 3 telephone interviews, women were asked whether any kind of painkillers, available over-the-counter or via prescription, had been taken. If women answered “yes,” they were asked to identify the type of drug(s) from a list of the 44 most common types, including acetaminophen as a mono or combination drug. Further questions gave respondents the option to report use of painkillers or drugs not specified in the list, as well as to record intake or drugs used to treat rheumatic diseases, infections, fever, or inflammation. Further, the women were asked to specify in which gestational weeks they used each of the listed drugs. Because the women did not indicate the dose of the painkillers used, cumulative weeks of use indicated exposure level in this study.

### Outcome measures

Information on pubertal development was collected by a translated version of a questionnaire developed by the British Avon Longitudinal Study of Parents and Children (ALSPAC) (26). The questionnaire is available at http://www.bsig.dk. The participants were asked every sixth month to report whether they had experienced the following pubertal milestones: axillary hair growth (yes/no), acne (yes/no), pubic hair growth and breast or genital development according to Tanner staging (1–5), first ejaculation (yes/no), first menstrual bleeding (yes/no), voice break (yes (sometimes), yes (definitive change), no, don’t know). If they answered “yes” to first ejaculation (boys) or first menstrual bleeding (girls), they were asked to provide age by year and months. Questions on pubic hair growth and breast or genital growth were guided by illustrations and explanatory texts for each of the Tanner stages (24, 25).

### Covariates

Directed acyclic graphs were used to identify potential confounding factors a priori (27). The following potential confounders were prepregnancy body mass index, alcohol consumption in the first trimester, smoking in the first trimester, time to pregnancy, highest social class of parents, maternal age at menarche, maternal age at delivery, and parity. In order to partly control for confounding by indication, 3 variables describing conditions that might induce use of acetaminophen during pregnancy were further included in our models. These were fever during pregnancy, muscle or joint disease during pregnancy, and inflammation or infection during pregnancy. The categorization of the included potential confounders can be found in Table 1.

**Table 1.** Maternal Characteristics According to Sons’ and Daughters’ Exposure to Acetaminophen at Least Once During Gestation, the Puberty Cohort (*n =* 15,822), Denmark, 2012–2017

Covariates^a^	Exposure to Acetaminophen				Total No.	Missing	
	No		Yes				
	No.	%	No.	%		No.	%
Total	7,216	45.6	8,606	54.4	15,822		
Prepregnancy BMI^b,c^	23.4 (4.4)		24.2 (4.7)		23.8 (4.6)	217	1.4
Alcohol consumption per week in first trimester, units						22	0.1
0	3,853	53.3	4,310	50.1	8,163		
>0–1	2,179	30.3	2,751	32.0	4,930		
>1–3	818	11.4	1,084	12.6	1,902		
>3	352	4.9	453	5.3	805		
No. of cigarettes daily in first trimester^c^	1.8 (4.2)		2.2 (4.5)		2.0 (4.3)	53	0.3
Time to pregnancy, including ART, months						44	0.3
0	1,380	19.2	1,656	19.3	3,036		
1–2	1,331	18.5	1,621	18.9	2,952		
3–5	1,158	16.1	1,398	16.3	2,556		
6–12	97	13.5	1,067	12.4	2,038		
>12	539	7.5	703	8.2	1,242		
Use of ART	702	9.8	762	8.9	1,464		
Not planned	1,113	15.5	1,377	16.0	2,490		
Highest social class of parents						31	0.2
High-grade professional	1,706	23.7	1,984	23.1	3,690		
Low-grade professional	2,375	33.0	2,821	32.8	5,196		
Skilled worker	2,011	27.9	2,342	27.3	4,353		
Unskilled worker	924	12.8	1,225	14.3	2,149		
Student	145	2.0	167	1.9	312		
Economically inactive	42	0.6	49	0.6	91		
Maternal age at menarche						123	0.8
Earlier than peers	1,724	24.1	2,288	26.7	4,012		
Same time as peers	4,099	57.4	4,891	57.2	8,990		
Later than peers	1,322	18.5	1,375	16.1	2,697		
Maternal age at delivery, years^c^	30.7 (4.4)		30.6 (4.4)		30.6 (4.4)	6	0.1
Parity						0	0.0
First child	3,829	53.1	4,138	48.1	7,967		
Second or later child	3,387	46.9	4,468	51.9	7,855		
Fever during pregnancy						23	0.1
No	5,657	78.5	5,758	67.0	11,415		
Yes	1,551	21.5	2,833	33.0	4,384		
Muscle or joint disease during pregnancy						7	0.1
No	6,247	86.6	7,038	81.8	13,285		
Yes	965	13.4	1,565	18.2	2,530		
Inflammation or infection during pregnancy						261	1.6
No	6,205	88.3	7,048	82.6	13,253		
Yes	825	11.7	1,483	17.4	2,308		

Abbreviations: ART, assisted reproductive techniques; BMI, body mass index.

^a^ Numbers within each categorized covariate might not necessarily add up to the total number of participants due to missing data.

^b^ Weight (kg)/height (m)^2^.

^c^ Values are expressed as mean (standard deviation).

Information on parity and maternal age was retrieved from the Danish Medical Birth Register, and socioeconomic status, classified based on the International Standard Class of Occupation and Education codes (ISCO-88 and ISCED), was retrieved from Statistics Denmark. Information on the remaining confounders was available through the telephone interviews in DNBC.

### Statistical analysis

The participants in the Puberty Cohort received the questionnaires half-yearly, which makes data on pubertal milestones censored. The statistical analyses were therefore carried out using a regression model for censored and normally distributed time-to-event data (intreg in Stata (StataCorp LLC, College Station, Texas)) to estimate mean monthly differences between exposure groups in age at attaining the pubertal milestones. The regression model is able to account for the left, right, or interval censoring of data assuming that the underlying distribution of the timing of a pubertal milestone is normal.

The assumption of normality was assessed by comparison of the stepwise cumulative incidence function and the cumulative incidence function based on the normal distribution using the R package icenReg (R Foundation for Statistical Computing, Vienna, Austria). The normal distribution fit the time of attained pubertal milestones very well. We also compared the nonparametric distribution to other waiting time distributions (Weibull, logistic, and log-normal), but the normal distribution gave the best fit. Residuals were calculated by subtracting the model mean to the left and right interval endpoints. The assumption of normality of these residuals was checked by a similar comparison further stratified by levels of included covariates.

We analyzed associations between intake of acetaminophen at least once during pregnancy and pubertal milestones using data from children with no recorded exposure during pregnancy as the unexposed referent. In the next analysis, age at attaining the pubertal milestones among children exposed in a cumulative number of weeks (1–2, 3–12, >12 weeks) was compared with age at attaining the milestones among the unexposed, and test for trend was conducted by fitting the number of weeks of exposure as a continuous variable. We further assessed timing of exposure comparing exposure only during the first (weeks 1–12 of gestation), second (weeks 13–24 of gestation), or third (week 25 of gestation until delivery) trimester and exposure in any 2 or all 3 trimesters with the unexposed.

The models were fitted with robust standard errors to account for clustering of siblings (*n =* 464 mothers) and sample as well as selection weights to address the sampling strategy and selective participation in the Puberty Cohort. The sampling weights estimate the inverse probability of being sampled for each individual in the Puberty Cohort according to the 28 different sampling frames. The selection weights estimate the inverse probability of participation according to a directed acyclic graph used to identify potential factors associated with participation in the Puberty Cohort a priori. Information on these factors, primarily related to socioeconomic conditions, were available as described above.

We conducted 2 subanalyses to further address confounding by indication. First, the analyses were restricted to mothers who did not report any of the 3 mentioned indications. Second, we performed analyses with the exclusion of mothers who took acetaminophen for more than 24 weeks of gestation (3%), because heavy use of acetaminophen during pregnancy might be more common in women suffering from chronic diseases causing pain. Because gestational weight gain might act as a potential confounder by altering infant size at birth (28), we repeated our analyses including gestational weight gain as an additional continuous covariate. To address the risk of type-1 errors due to multiple testing, we performed a test for the overall association with all puberty markers using the Huber-White robust variance estimation applied on the univariate marker models, which accounts for the correlations structure between the pubertal markers (29, 30). These subanalyses were performed for cumulative weeks of exposure.

Statistical analyses were conducted using Stata/MP, version 13.1 (StataCorp LLC), and R x64, version 3.3.1 (R Foundation for Statistical Computing).

### Ethical approval

The Committee for Biomedical Research Ethics in Denmark has approved data collection in DNBC ((KF) 01-471/94). A written informed consent was obtained from mothers upon recruitment covering both mother’s and offspring’s participation until the children turned 18 years of age. The present study was approved by the steering committee of DNBC (2012-04 and 2015-47) and the Danish Data Protection Agency (2012-41-0379 and 2015-57-0002).

## RESULTS

Of the 22,439 children invited to participate in the Puberty cohort, 15,822 (approximately 71%), 7,697 boys and 8,125 girls, have returned at least 1 and up to 11 questionnaires each (Figure 1). Use of acetaminophen was not related to participation in the Puberty Cohort. The responders were, by March 2017, between 14 and 17 years of age, and 4,317 had reached full sexual maturity (27%). Among the 15,822 participants, 8,606 (54%) were exposed to acetaminophen at least once during gestation. Table 1 shows that prepregnancy body mass index, parity, and reports of the 3 potential indications were higher in mothers with any gestational intake of acetaminophen. In addition, maternal smoking and alcohol intake during the first trimester were more frequent in this group.

**Figure 2.**
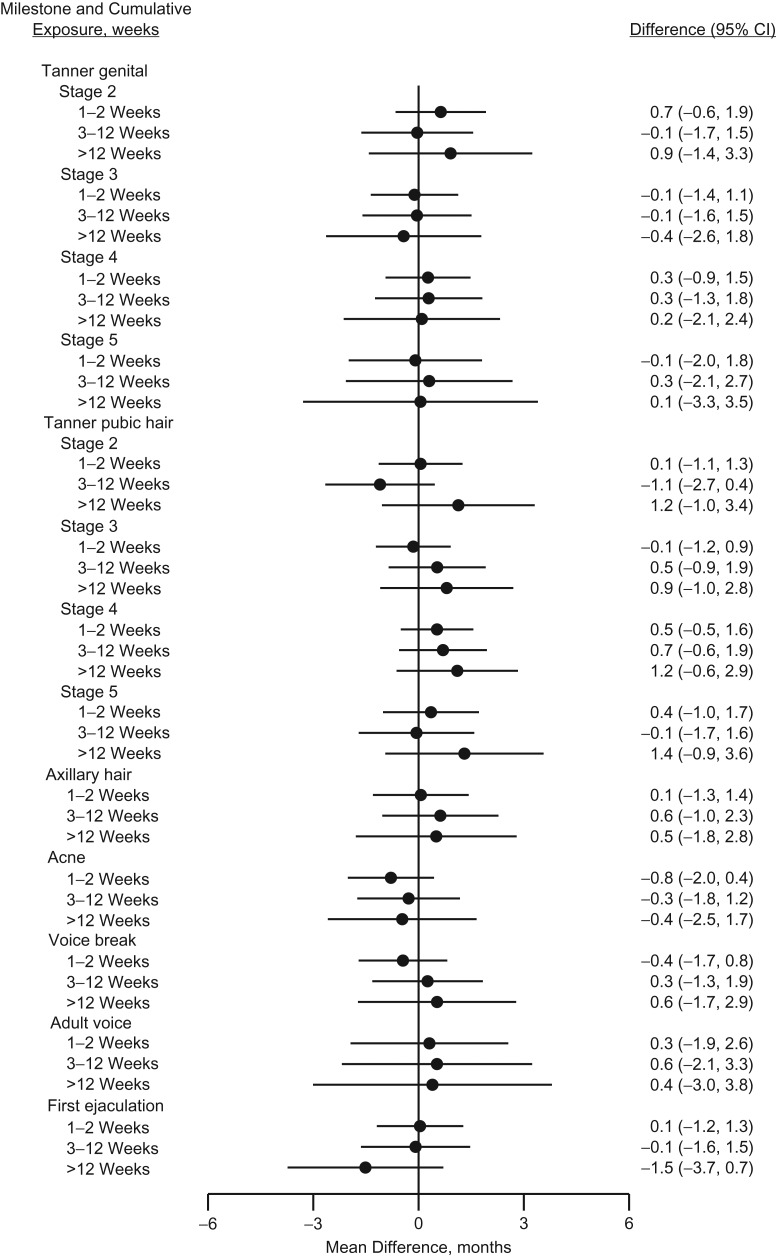
Adjusted mean differences (months, 95% confidence intervals (CIs)) in age at attaining various pubertal milestones in boys according to number of weeks with exposure to acetaminophen during gestation, the Puberty Cohort, Denmark, 2012–2017.

**Figure 3.**
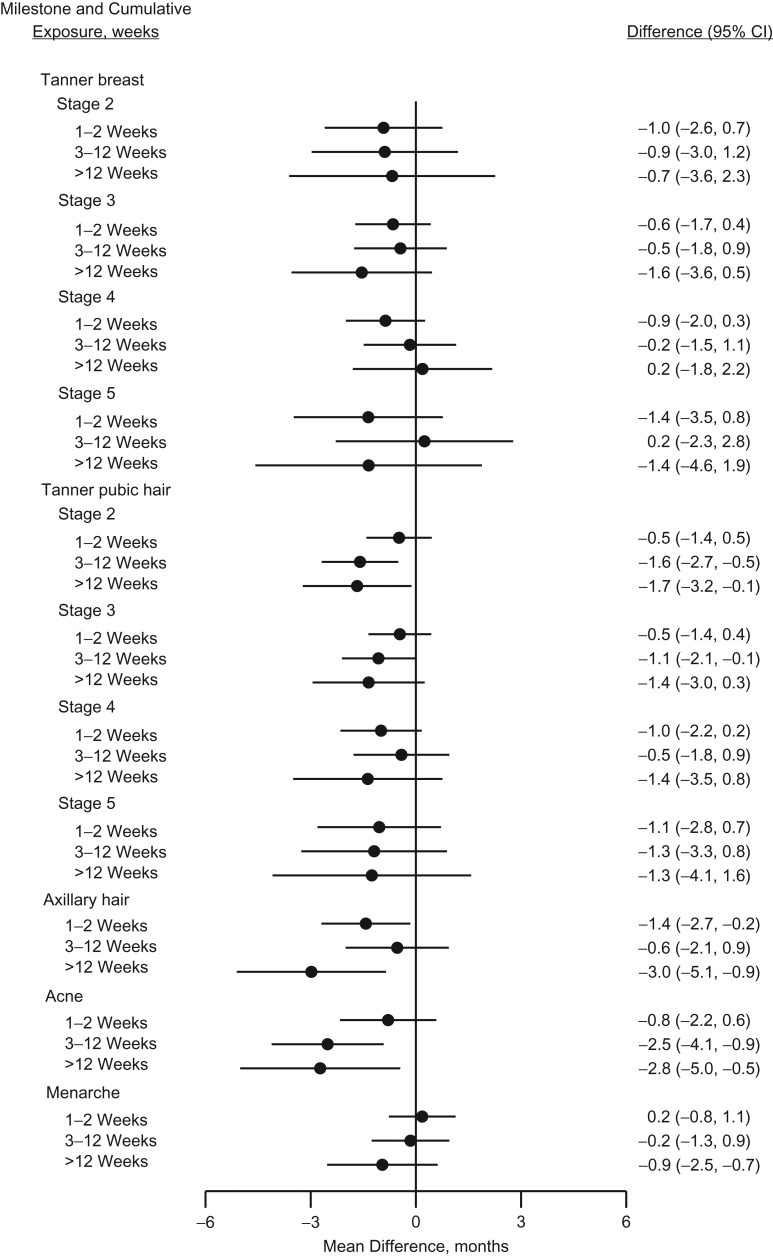
Adjusted mean differences (months, 95% confidence intervals (CIs)) in age at attaining various pubertal milestones in girls according to number of weeks with exposure to acetaminophen during gestation, the Puberty Cohort, Denmark, 2012–2017.

Table 2 presents the mean ages in years at attaining various male and female pubertal milestones for an unexposed reference person as well as the mean monthly differences between those who were and who were not exposed to acetaminophen at least once during fetal life. The crude estimates suggested a tendency towards earlier age at pubertal timing in exposed boys, which was attenuated in the adjusted analyses. In exposed girls, a more consistent tendency towards slightly earlier age at pubertal timing was observed. The tendency was, however, most pronounced for axillary hair growth, with −1.2 (95% confidence interval (CI): −2.2, −0.2), and occurrence of acne, with −1.7 (95% CI: −2.7, −0.6) months earlier age at attainment. Analyses of cumulative number of weeks with exposure to acetaminophen during pregnancy are presented in Figures 2 and 3 (and Web Table 1, available at https://academic.oup.com/aje) as adjusted monthly differences between exposure groups. We observed no strong indication of a pattern with increasing number of weeks of exposure in boys, although all of the Tanner pubic hair stages were to some extent shifted towards later age at attainment in boys exposed for more than 12 weeks. A somewhat consistent pattern was observed among girls, associating longer duration of intrauterine acetaminophen exposure with earlier age at attaining almost all studied markers of pubertal development, but with wide confidence intervals. The magnitudes of the observed associations were largest for stage 2 of Tanner pubic hair (difference, months = −1.7, 95% CI: −3.2, −0.1), axillary hair growth (difference, months = −3.0, 95% CI: −5.1, −0.9), and occurrence of acne (difference, months = −2.8, 95% CI: −5.0, −0.5) in the group of girls exposed to acetaminophen for more than 12 weeks.

**Table 2.** Mean Differences in Age at Attaining Various Pubertal Milestones According to Exposure to Acetaminophen at Least Once During Gestation, the Puberty Cohort, Denmark, 2012–2017

Pubertal Milestone	No. of Persons^a^	Exposure to Acetaminophen				
		No^b^		Yes^c^		
		Mean Age, years	95% CI	Crude	Adjusted^d^	
				Mean Difference	Mean Difference	95% CI
Boys						
Tanner stage, genitals						
Stage 2	7,469	11.2	10.5, 11.9	−0.3	0.0	−1.0, 1.0
Stage 3	7,469	12.7	11.9, 13.5	−0.7	−0.2	−1.2, 0.8
Stage 4	7,469	13.7	12.0, 14.4	−0.4	0.1	−0.9, 1.0
Stage 5	7,469	15.2	14.3, 16.0	−0.4	0.3	−1.2, 1.8
Tanner stage, pubic hair						
Stage 2	7,473	11.7	11.0, 12.4	−0.7	−0.3	−1.2, 0.7
Stage 3	7,473	12.7	12.0, 13.4	−0.5	0.1	−0.7, 1.0
Stage 4	7,473	13.7	13.2, 14.3	0.1	0.6	−0.2, 1.4
Stage 5	7,473	14.9	14.4, 15.4	−0.3	0.2	−0.8, 1.3
Axillary hair	7,478	13.5	12.7, 14.3	−0.2	0.4	−0.7, 1.4
Acne	7,478	11.5	10.9, 12.2	−0.8	−0.3	−1.3, 0.6
Voice break	7,274	13.1	12.4, 13.8	−0.5	0.1	−0.9, 1.1
Adult voice	7,274	14.4	13.1, 15.6	−0.4	0.5	−1.2, 2.2
First ejaculation	7,465	13.5	12.9, 14.2	−0.7	−0.3	−1.2, 0.7
Girls						
Tanner stage, breast						
Stage 2	7,888	10.7	9.9, 11.5	−1.3	−0.7	−2.0, 0.7
Stage 3	7,888	12.1	11.5, 12.7	−1.2	−0.6	−1.4, 0.3
Stage 4	7,888	13.4	12.7, 14.1	−1.0	−0.3	−1.3, 0.6
Stage 5	7,888	16.0	15.1, 17.0	−1.5	−0.4	−2.1, 1.2
Tanner stage, pubic hair						
Stage 2	7,889	11.4	10.9, 11.9	−1.0	−0.7	−1.5, 1.2
Stage 3	7,889	12.9	12.5, 13.3	−0.9	−0.6	−1.3, 0.1
Stage 4	7,889	13.8	13.4, 14.3	−1.0	−0.7	−1.6, 0.2
Stage 5	7,889	15.7	14.9, 16.5	−1.3	−0.8	−2.1, 0.6
Axillary hair	7,894	12.2	11.6, 12.9	−1.7	−1.2	−2.2, −0.2
Acne	7,894	11.9	11.4, 12.5	−2.1	−1.7	−2.7, −0.6
Menarche	7,886	13.4	12.9, 13.8	−0.7	−0.1	−0.8, 0.7

Abbreviations: BMI, body mass index; CI, confidence interval.

^a^ Number of persons included in analysis for each milestone.

^b^ Mean age in years at attaining pubertal milestones for a given reference person with no exposure to acetaminophen: prepregnancy BMI of 18.5 (calculated as weight (kg)/height (m)^2^), 0 alcohol units per week in first trimester, 0 cigarettes daily in first trimester, 0 months time to pregnancy, highest social class of parents was high-grade professional, maternal age of menarche same as peers, maternal age at delivery of 30.7 years, parity: first child, no fever during pregnancy, no muscle or joint disease during pregnancy, and no inflammation or infection during pregnancy.

^c^ Mean difference (months) at attaining milestone between unexposed and exposed.

^d^ Adjusted for prepregnancy BMI, alcohol units per week in first trimester, daily number of cigarettes in first trimester, time to pregnancy, highest social class of parents, maternal age of menarche, maternal age at delivery, parity, fever during pregnancy, muscle or joint disease during pregnancy, and inflammation or infection during pregnancy.

**Figure 4.**
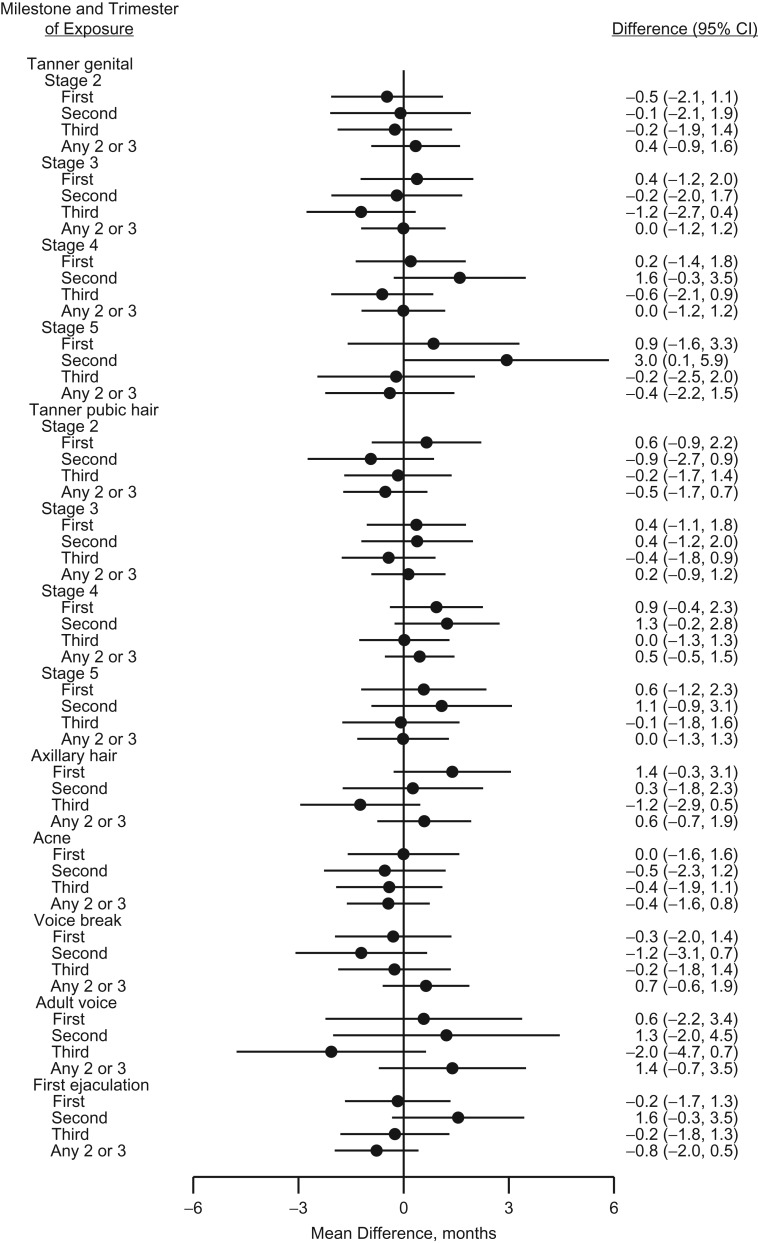
Adjusted mean differences (months, 95% confidence intervals (CIs)) in age at attaining various pubertal milestones in boys according to trimester of exposure to acetaminophen during gestation, the Puberty Cohort, Denmark, 2012–2017.

**Figure 5.**
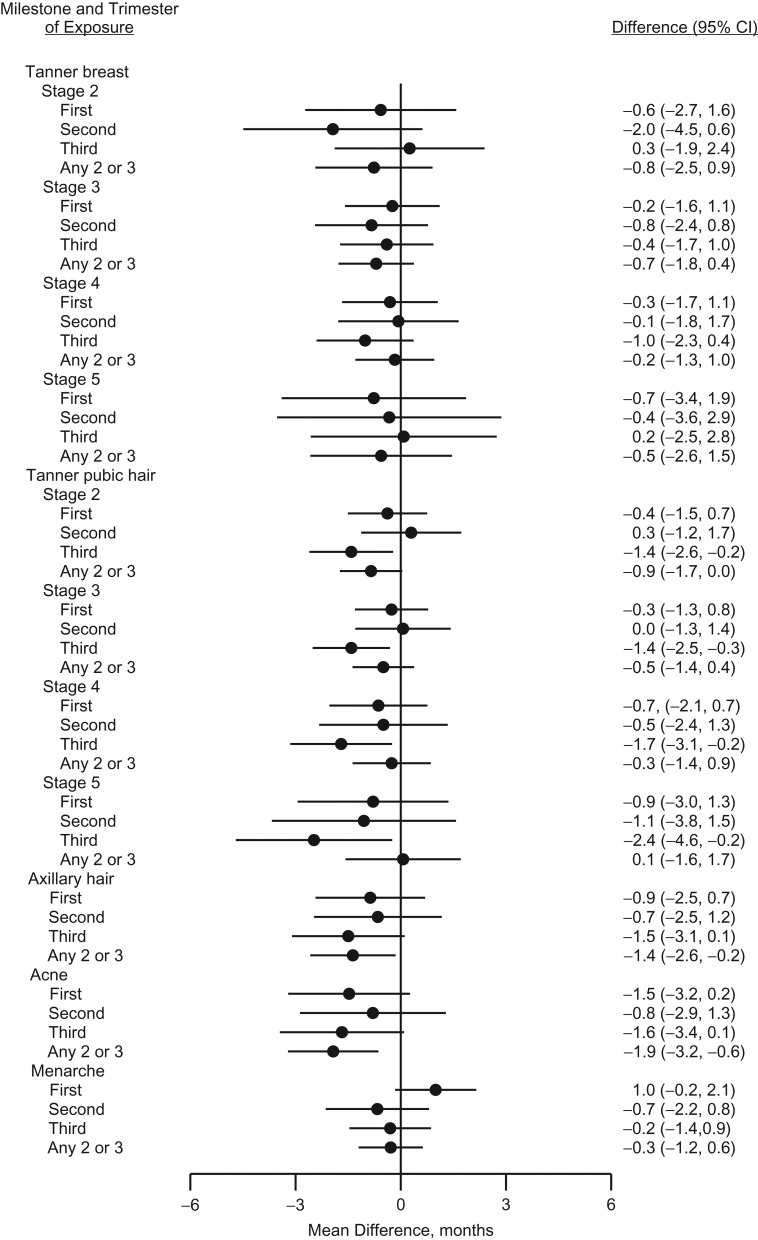
Adjusted mean differences (months, 95% confidence intervals (CIs)) in age at attaining various pubertal milestones in girls according to trimester of exposure to acetaminophen during gestation, the Puberty Cohort, Denmark, 2012–2017.

The analyses on timing of exposure by trimester showed no specific pattern in boys (Figure 4 and Web Table 2). In girls, breast development was independent of the specific timing of exposure, whereas pubic hair development and to a lesser degree axillary hair growth and occurrence of acne were skewed towards earlier age at attainment in girls with exposure in one of the trimesters. These tendencies were most pronounced in girls exposed only in the third trimester (Figure 5 and Web Table 2).

In the analyses restricted to mothers who did not report any of the 3 conditions associated with acetaminophen intake, results were similar to those presented for the binary and trimester-specific exposure to acetaminophen. The tendency towards earlier age at female pubic hair development, however, strengthened in the analyses with cumulative weeks of exposure (data not shown). Analyses with exclusion of heavy acetaminophen users did not change our results substantially. In the analyses with cumulative weeks of exposure, however, female axillary hair growth and occurrence of acne were further shifted towards earlier age at attainment among those who were highly exposed (more than 12 weeks of gestation) (data not shown). Our subanalyses with additional adjustment for gestational weight gain did not change the results (data not shown). Using robust variance estimation to perform a single test, cumulative number of weeks with exposure was associated with earlier female puberty timing (*P* = 0.04).

## DISCUSSION

Results from our population-based cohort study suggest that intrauterine exposure to acetaminophen might be associated with slightly earlier attainment of almost all studied female markers of pubertal development, although most pronounced for pubic hair growth, axillary hair growth, and occurrence of acne, but with wide confidence intervals. On the other hand, estimates for boys were randomly scattered around the null hypothesis, indicating limited if any evidence for an association between intrauterine exposure to acetaminophen and male pubertal development. These findings were supported by a test of the overall association between exposure and all puberty markers. We were not able to identify specific vulnerable exposure windows. Our results suggest that those exposed to acetaminophen during fetal life might exhibit sex-specific endocrine disruptive effects in relation to pubertal development.

Prior studies have mainly addressed whether in utero exposure to acetaminophen interferes with neurodevelopment and cognition (2, 4, 31–37), asthma (38), markers of short-term reproductive health, such as risk of some congenital anomalies (9, 17–22), and differences in anogenital distance (15, 16). Intrauterine exposure to acetaminophen might interfere with important fetal developmental processes regulated by prostaglandins (12, 14, 39) and/or exert direct inhibitory effects on fetal testosterone production (9, 10). However, it remains unknown to what extent such potential effects are translatable to humans and might induce long-lasting effects on the complex neuroendocrine system that regulates pubertal development and which parts of the regulatory system might be susceptible to acetaminophen exposure.

Puberty is initiated by a reactivation of the hypothalamic-pituitary-gonadal axis that stimulates androgen production, causing testicular and pubic hair growth in boys, and stimulates ovarian estrogen production, causing breast enlargement and menarche in girls. Girls’ pubic and axillary hair growth are initially regulated by androgens from the adrenal glands, and at later stages of puberty, hair growth becomes hypothalamic-pituitary-gonadal-dependent (40, 41). Earlier age at onset of pubic and axillary hair growth in girls, as seen in our study, might suggest that acetaminophen interferes with peripheral androgen production rather than inducing hypothalamic-pituitary-gonadal axis dysfunction. However, if acetaminophen also exerts peripheral antiandrogenic effects, lack of androgen would be expected to delay rather than accelerate puberty in girls.

Major strengths of the study are the high participation rate and the use of a variety of different markers of pubertal development. Further, information on exposure and pubertal development was collected close to the time of the event (23, 42). The participants ranked their current pubertal stage by a wide range of markers every 6 months beginning at age 11 years. A large proportion of the boys and girls had already experienced some of the early puberty stages at inclusion. Our regression model was, however, able to account for the censoring of data assuming that the underlying distribution of the timing of pubertal milestones is normal. A recent consensus paper strongly recommended the use of these types of regression models in longitudinal studies with repeated measures of pubertal development (43).

We used self-administrated questionnaires, including Tanner rating scales, to assess pubertal development. Although studies on the reliability of self-assessment of pubertal development using Tanner staging have been conflicting (44–47), any potential misclassification would most likely be nondifferential in this study. Further, self-reporting of pubertal development serves as a cost- and timing-savings tool in large longitudinal cohorts such as the Puberty Cohort that do not pose the same strong risk of selection bias compared with clinical examinations.

Information on use of acetaminophen was collected prior to outcome occurrence, including details on timing and duration of use. Lack of information and recall bias are therefore expected to be independent of later pubertal development. We used time of interview to assign trimester-specific exposure when mothers stated that they took acetaminophen but were unable to specify timing of use. In addition, 2,357 interviewees (15%) were unable to identify intake on a weekly basis, thereby introducing some uncertainty to the analyses on cumulative exposure. Moreover, the grouping of number of weeks of exposure was a crude indicator for cumulative duration of exposure, because it does not take into account the frequencies of use. This misclassification would often lead to an underestimation of the effect sizes.

Similarly, we were not able to account for the frequencies of use in the analyses with timing of exposure. Further, by combining intake of acetaminophen in any 2 or all 3 trimesters into one category, effects of cumulative and time-specific exposure to acetaminophen might be difficult to separate. We believe, however, that by comparing children with no exposure to children with exposure in one specific trimester, we provide some insight into potential vulnerable exposure windows.

We used inverse probability weighting to account for the sampling procedure and nonresponse and controlled for several potential confounders. Use of acetaminophen was, as expected, more common in pregnant women who reported episodes of inflammation or infection, fever, or musculoskeletal disorders. In subanalyses restricted to women with no reports of these indications, cumulative number of weeks with exposure was more strongly associated with earlier age at attaining pubic hair development, especially for children with more than 12 weeks of exposure. This finding could be partially explained by similar indications among this group of mothers, because these indications were slightly associated with earlier puberty timing in our data. A prolonged use of acetaminophen during pregnancy is more common in women suffering from chronic diseases such as rheumatic diseases, but we are not aware of studies that have investigated whether this chronic disease is associated with pubertal development. However, exclusion of heavy users of acetaminophen, defined as use for more than 24 weeks of gestation (3%), did not change our results substantially. Still, we cannot exclude the possibility that residual confounding by indication or unmeasured confounding factors might serve as alternative explanations, although that would require that these factors are also strong risk factors for earlier pubertal development in girls. A potential confounding factor could be unmeasured, poor, family-related lifestyle behavior that increased maternal intake of acetaminophen and accelerated pubertal timing by increasing the risk of high childhood body mass index. To examine the potential impact of residual confounding by indication from conditions for long-term use and unmeasured confounding from, for example, poor overall health, we conducted a multidimensional bias analysis using the approach suggested by VanderWeele and Arah (48). Even after applying realistic scenarios for female axillary hair growth, the impact of potential bias was minimal. Description and results of this analysis are found in the Web material (Web Appendix 1 and Web Table 3).

In conclusion, our study suggests a tendency towards earlier female pubertal development with increasing number of weeks of intrauterine exposure to acetaminophen but seems to show no strong associations, if any, with male pubertal development. The results suggest that acetaminophen might interfere with prenatal, sex-specific developmental processes that lead to long-term effects on pubertal development. Although the observed shifts in female puberty timing were minor, these findings could signal other endocrine-related changes that might have later reproductive and health disadvantages, as suggested by others (49–52).

## Supplementary Material

Web Material

## Abbreviations

CI: confidence interval
DNBC: Danish National Birth Cohort.
