# Supplementary material for: Acetaminophen (Paracetamol) Exposure During Pregnancy and Pubertal Development in Boys and Girls From a Nationwide Puberty Cohort
Source: Am J Epidemiol. 2018 Sep 7;188(1):34–46. doi: 10.1093/aje/kwy193 (PMC6321810; doi:10.1093/aje/kwy193)
Supplement: Web Material [file kwy193_ernst_web_material_final.pdf]

WEB MATERIAL

Acetaminophen (Paracetamol) Exposure During Pregnancy and Pubertal Development in Boys and Girls from a Nationwide Puberty Cohort

Andreas Ernst*, Nis Brix, Lea L. B. Lauridsen, Jørn Olsen, Erik T. Parner, Zeyan Liew, Lars H. Olsen, and Cecilia H. Ramlau-Hansen

Table of content

Web Appendix 1: *Multidimensional Bias Analysis of the Potential Impact of Unmeasured Confounding*

Web Table 1: *Mean Differences (Months, 95% CI) in Age at Attaining Various Pubertal Milestones According to Number of Weeks with Exposure to Acetaminophen during Pregnancy Compared to Unexposed, the Puberty Cohort, Denmark, March 2017*

Web Table 2: *Mean Differences (Months, 95% CI) in Age at Attaining Various Pubertal Milestones According to Trimester Specific Exposure to Acetaminophen during Pregnancy Compared to Unexposed, the Puberty Cohort, Denmark, March 2017*

Web Table 3: *Multidimensional Bias Analysis of the Association between Intrauterine Exposure to Acetaminophen (0 Weeks vs. >12 Weeks) and Age at Attaining Female Axillary Hair Growth.*

Web Appendix 1: Multidimensional Bias Analysis of the Potential Impact of Unmeasured Confounding

To explore the potential impact of unmeasured confounding in this study on intrauterine exposure to acetaminophen and pubertal development, we conducted a multidimensional bias analysis using the simplified approach recently suggested by VanderWeele TJ and Arah OA (1). This approach assumes that the unmeasured confounding variable (U) is binary and has a constant effect on the outcome across all strata of treatment and covariates. Further, it assumes a constant prevalence difference of the confounder between exposed and unexposed in all strata of the included covariates.

According to Table 1. in the manuscript, the maximum prevalence differences between unexposed and exposed at least once during pregnancy approximated 5 %-points across all covariates except the three indications for use (fever during pregnancy, muscle or joint disease during pregnancy or inflammation or infection during pregnancy). For the three indications, the maximum prevalence difference was approximately 11.5 %-points ("Yes" to fever during pregnancy). Based on these numbers, we defined one sensitivity parameter, *a*, as the prevalence difference of U between unexposed and exposed and considered three different levels of *a*: 5 %, 10 % and 20 %. Further, we defined a second sensitivity parameter, *b*, as the mean monthly change of U on age at attaining the pubertal milestones.
A prevalence difference, *a*, of 20 % is rather high and would most likely relate to potential unmeasured confounding by indication for use of acetaminophen e.g. by migraine. We do not expect conditions like migraine to be very strong risk factors for puberty timing. Other unmeasured confounders related to e.g. poor life style behavior may have a stronger effect on puberty timing than unmeasured confounding by indication. However, these unmeasured confounders will probably be less unevenly distributed between unexposed and exposed, especially taken into consideration the covariates already included in our models. Based on these considerations, we evaluated the following values of *b*: 3 months and 6 months. Given the assumptions stated above (1), subtracting the product of *a* x *b* for each combination of *a* and *b* from the original estimates and their 95 % confidence intervals, we would obtain the bias-corrected counterparts.

For this purpose, we performed the bias analysis on the most significant result from the models with cumulative weeks of intrauterine exposure to acetaminophen. As seen in Web Table 2, girls exposed to acetaminophen for more than 12 weeks seemed to experience axillary hair growth −3.0 (95 % CI: −5.1, −0.9) months earlier when compared to unexposed girls. The results from the analyses can be found in the Web Table 3.

If we consider a potential harmful unmeasured confounder/residual confounder e.g. related to socioeconomic status or poor lifestyle behavior, we would expect this confounder to be 5 or 10 % more prevalent among users of acetaminophen and to accelerate puberty timing by 6 months. In this scenario, the bias-corrected estimate would be −2.7 (−4.8, −0.6) or −2.4 (−4.5, −0.3) (left lower corner of the Web Table 3). On the other hand, considering a potential unmeasured confounder related to indication for use such as migraine, we would expect a higher prevalence difference of 20 %, but a smaller effect on the timing of puberty timing such as −3 months. In this scenario, the bias-corrected estimate would be −2.4 (−4.5, −0.3).

In conclusion, even though we apply relatively realistic scenarios in this bias analysis, the bias-corrected estimates remain significant as indicated in Web Table 3.

References

1. Vanderweele TJ, Arah OA. Bias formulas for sensitivity analysis of unmeasured confounding for general outcomes, treatments, and confounders. *Epidemiology (Cambridge, Mass)* 2011;22(1):42-52.

Web Table 1. Mean Differences (Months, 95% CI) in Age at Attaining Various Pubertal Milestones According to Number of Weeks with Exposure to Acetaminophen during Pregnancy Compared to Unexposed, the Puberty Cohort, Denmark,

		Total number of weeks with exposure to acetaminophen during pregnancy									
		1 - 2 weeks			3 - 12 weeks			>12 weeks			Test for trend[b]
		Crude	Adjusted[a]		Crude	Adjusted[a]		Crude	Adjusted[a]		
Pubertal milestone	No. of persons[c]	Mean diff.	Mean diff.	95 % CI	Mean diff.	Mean diff.	95 % CI	Mean diff.	Mean diff.	95 % CI	*P*-value
**Boys**											
Tanner stages - Genitals											
Stage 2	6,348	0.4	0.7	−0.6, 1.9	−0.4	−0.1	−1.7, 1.5	0.6	0.9	−1.4, 3.3	0.56
Stage 3	6,348	−0.5	−0.1	−1.4, 1.1	−0.5	−0.1	−1.6, 1.5	−1.1	−0.4	−2.6, 1.8	0.55
Stage 4	6,348	−0.2	0.3	−0.9, 1.5	−0.4	0.3	−1.3, 1.8	−0.5	0.2	−2.1, 2.4	0.74
Stage 5	6,348	−0.5	−0.1	−2.0, 1.8	−0.4	0.3	−2.1, 2.7	−0.7	0.1	−3.3, 3.5	0.95
Tanner stages - Pubic hair											
Stage 2	6,352	−0.4	0.1	−1.1, 1.3	−1.6	−1.1	−2.7, 0.4	0.7	1.2	−1.0, 3.4	0.69
Stage 3	6,352	−0.7	−0.1	−1.2, 0.9	−0.2	0.5	−0.9, 1.9	0.2	0.9	−1.0, 2.8	0.40
Stage 4	6,352	0.1	0.5	−0.5, 1.6	0.3	0.7	−0.6, 1.9	0.6	1.2	−0.6, 2.9	0.54
Stage 5	6,352	−0.0	0.4	−1.0, 1.7	−0.6	−0.1	−1.7, 1.6	0.6	1.4	−0.9, 3.6	0.37
Axillary hair	6,357	−0.3	0.1	−1.3, 1.4	0.0	0.6	−1.0, 2.3	−0.2	0.5	−1.8, 2.8	0.92
Acne	6,357	−1.2	−0.8	−2.0, 0.4	−0.8	−0.3	−1.8, 1.2	−1.0	−0.4	−2.5, 1.7	0.79
Voice break	6,183	−0.9	−0.4	−1.7, 0.8	−0.5	0.3	−1.3, 1.9	−0.2	0.6	−1.7, 2.9	0.50
Adult voice	6,183	−0.5	0.3	−1.9, 2.6	−0.4	0.6	−2.1, 3.3	−0.9	0.4	−3.0, 3.8	0.90
First ejaculation	6,344	−0.3	0.1	−1.2, 1.3	−0.6	−0.1	−1.6, 1.5	−2.0	−1.5	−3.7, 0.7	0.31
**Girls**											
Tanner stages - Breast											
Stage 2	6,720	−1.4	−1.0	−2.6, 0.7	−1.7	−0.9	−3.0, 1.2	−2.0	−0.7	−3.6, 2.3	0.68
Stage 3	6,720	−1.0	−0.6	−1.7, 0.4	−1.3	−0.5	−1.8, 0.9	−2.8	−1.6	−3.6, 0.5	0.28
Stage 4	6,720	−1.2	−0.9	−2.0, 0.3	−1.1	−0.2	−1.5, 1.1	−1.1	0.2	−1.8, 2.2	0.36
Stage 5	6,720	−2.0	−1.4	−3.5, 0.8	−1.1	0.2	−2.3, 2.8	−3.4	−1.4	−4.6, 1.9	0.68
Tanner stages - Pubic hair											
Stage 2	6,721	−0.7	−0.5	−1.4, 0.5	−1.9	−1.6	−2.7, −0.5	−2.1	−1.7	−3.2, −0.1	0.03
Stage 3	6,721	−0.7	−0.5	−1.4, 0.4	−1.5	−1.1	−2.1, −0.1	−2.0	−1.4	−3.0, 0.3	0.10
Stage 4	6,721	−1.1	−1.0	−2.2, 0.2	−1.0	−0.5	−1.8, 0.9	−2.0	−1.4	−3.5, 0.8	0.37
Stage 5	6,721	−1.4	−1.1	−2.8, 0.7	−2.1	−1.3	−3.3, 0.8	−2.1	−1.3	−4.1, 1.6	0.34
Axillary hair	6,725	−1.9	−1.4	−2.7, −0.2	−1.2	−0.6	−2.1, 0.9	−3.9	−3.0	−5.1, −0.9	0.08
Acne	6,725	−1.2	−0.8	−2.2, 0.6	−3.0	−2.5	−4.1, −0.9	−3.5	−2.8	−5.0, −0.5	0.02
Menarche	6,718	−0.2	0.2	−0.8, 1.1	−0.8	−0.2	−1.3, 0.9	−2.0	−0.9	−2.5, 0.7	0.39

March 2017

Abbreviations: CI, confidence interval

[a]Adjusted for pre-pregnancy body mass index, alcohol units per week in first trimester, daily number of cigarettes in first trimester, time to pregnancy, highest social class of parents, maternal age of menarche, maternal age at delivery, parity, fever during pregnancy, muscle or joint disease during pregnancy and inflammation or infection during pregnancy [b]Test for trend by number of weeks of exposure to acetaminophen during pregnancy as a continuous variable (0 – 40 weeks). 2-sided test.
[c]Number of persons included in adjusted analysis for each milestone

Web Table 2. Mean Differences (Months, 95% CI) in Age at Attaining Various Pubertal Milestones According to Trimester Specific Exposure to Acetaminophen during Pregnancy Compared to Unexposed, the Puberty Cohort, Denmark, March 2017

		Trimester specific exposure to acetaminophen during pregnancy											
		Only 1. trimester			Only 2. trimester			Only 3. trimester			Any 2 or all 3 trimester		
		Crude	Adjusted[a]		Crude	Adjusted[a]		Crude	Adjusted[a]		Crude	Adjusted[a]	
Pubertal milestone	No. of persons[b]	Mean diff.	Mean diff.	95 % CI	Mean diff.	Mean diff.	95 % CI	Mean diff.	Mean diff.	95 % CI	Mean diff.	Mean diff.	95 % CI
**Boys**													
Tanner stages - Genitals													
Stage 2	7,468	−0.7	−0.5	−2.1, 1.1	−0.6	−0.1	−2.1, 1.9	−0.6	−0.2	−1.9, 1.4	0.1	0.4	−0.9, 1.6
Stage 3	7,468	−0.0	0.4	−1.2, 2.0	−0.4	−0.2	−2.0, 1.7	−1.8	−1.2	−2.7, 0.4	−0.5	0.0	−1.2, 1.2
Stage 4	7,468	−0.3	0.2	−1.4, 1.8	1.5	1.6	−0.3, 3.5	−1.2	−0.6	−2.1, 0.9	−0.6	0.0	−1.2, 1.2
Stage 5	7,468	0.3	0.9	−1.6, 3.3	2.9	3.0	0.1, 5.9	−1.1	−0.2	−2.5, 2.0	−1.2	−0.4	−2.2, 1.5
Tanner stages - Pubic hair													
Stage 2	7,472	0.2	0.6	−0.9, 2.2	−1.4	−0.9	−2.7, 0.9	−0.6	−0.2	−1.7, 1.4	−1.0	−0.5	−1.7, 0.7
Stage 3	7,472	−0.1	0.4	−1.1, 1.8	−0.1	0.4	−1.2, 2.0	−1.1	−0.4	−1.8, 0.9	−0.5	0.2	−0.9, 1.2
Stage 4	7,472	0.6	0.9	−0.4, 2.3	1.0	1.3	−0.2, 2.8	−0.4	0.0	−1.3, 1.3	−0.1	0.5	−0.5, 1.5
Stage 5	7,472	0.3	0.6	−1.2, 2.3	1.0	1.1	−0.9, 3.1	−0.7	−0.1	−1.8, 1.6	−0.7	0.0	−1.3, 1.3
Axillary hair	7,477	0.9	1.4	−0.3, 3.1	0.3	0.3	−1.8, 2.3	−1.6	−1.2	−2.9, 0.5	−0.1	0.6	−0.7, 1.9
Acne	7,477	−0.3	0.0	−1.6, 1.6	−1.0	−0.5	−2.3, 1.2	−0.8	−0.4	−1.9, 1.1	−0.8	−0.4	−1.6, 0.8
Voice break	7,273	−0.7	−0.3	−2.0, 1.4	−1.3	−1.2	−3.1, 0.7	−0.8	−0.2	−1.8, 1.4	0.8	0.7	−0.6, 1.9
Adult voice	7,273	−0.2	0.6	−2.2, 3.4	1.0	1.3	−2.0, 4.5	−3.1	−2.0	−4.7, 0.7	0.4	1.4	−0.7, 3.5
First ejaculation	7,464	−0.7	−0.2	−1.7, 1.3	1.4	1.6	−0.3, 3.5	−0.7	−0.2	−1.8, 1.3	−1.2	−0.8	−2.0, 0.5
**Girls**													
Tanner stages - Breast													
Stage 2	7,888	−1.0	−0.6	−2.7, 1.6	−2.4	−2.0	−4.5, 0.6	−0.1	0.3	−1.9, 2.4	−1.8	−0.8	−2.5, 0.9
Stage 3	7,888	−0.8	−0.2	−1.6, 1.1	−0.9	−0.8	−2.4, 0.8	−0.6	−0.4	−1.7, 1.0	−1.6	−0.7	−1.8, 0.4
Stage 4	7,888	−1.1	−0.3	−1.7, 1.1	−0.2	−0.1	−1.8, 1.7	−1.2	−1.0	−2.3, 0.4	−1.1	−0.2	−1.3, 1.0
Stage 5	7,888	−1.7	−0.7	−3.4, 1.9	−0.5	−0.4	−3.6, 2.9	−0.5	0.2	−2.5, 2.8	−2.1	−0.5	−2.6, 1.5
Tanner stages - Pubic hair													
Stage 2	7,889	−0.8	−0.4	−1.5, 0.7	0.1	0.3	−1.2, 1.7	−1.5	−1.4	−2.6, −0.2	−1.1	−0.9	−1.7, 0.0
Stage 3	7,889	−0.7	−0.3	−1.3, 0.8	−0.1	0.0	−1.3, 1.4	−1.5	−1.4	−2.5, −0.3	−0.9	−0.5	−1.4, 0.4
Stage 4	7,889	−1.1	−0.7	−2.1, 0.7	−0.6	−0.5	−2.4, 1.3	−1.7	−1.7	−3.1, −0.2	−0.7	−0.3	−1.4, 0.9
Stage 5	7,889	−1.4	−0.9	−3.0, 1.3	−1.3	−1.1	−3.8, 1.5	−2.6	−2.4	−4.6, −0.2	−0.7	0.1	−1.6, 1.7
Axillary hair	7,894	−1.5	−0.9	−2.5, 0.7	−0.9	−0.7	−2.5, 1.2	−1.8	−1.5	−3.1, 0.1	−2.0	−1.4	−2.6, −0.2
Acne	7,894	−2.0	−1.5	−3.2, 0.2	−1.4	−0.8	−2.9, 1.3	−2.0	−1.6	−3.4, 0.1	−2.4	−1.9	−3.2, −0.6
Menarche	7,886	0.2	1.0	−0.2, 2.1	−0.8	−0.7	−2.2, 0.8	−0.5	−0.2	−1.4, 0.9	−1.0	−0.3	−1.2, 0.6

Abbreviations: CI, confidence interval

[a]Adjusted for pre-pregnancy body mass index, alcohol units per week in first trimester, daily number of cigarettes in first trimester, time to pregnancy, highest social class of parents, maternal age of menarche, maternal age at delivery, parity, fever during pregnancy, muscle or joint disease during pregnancy and inflammation or infection during pregnancy [b]Number of persons included in adjusted analysis for each milestone

Webtable 3. Multidimensional Bias Analysis of the Association between Intrauterine Exposure to Acetaminophen (0 Weeks vs. >12 Weeks) and Age at Attaining Female Axillary Hair Growth.

	Adjusted[c] mean difference (months, 95% CI) in age at attaining axillary hair growth between unexposed (0 weeks) and exposed (>12 weeks) girls									
	b[b] = −6 months		*b* = −3 months		*b* = 0 months		*b* = +3 months		*b* = +3 months	
a[a]	Age difference[d]	95 % CI	Age difference	95 % CI	Age difference	95 % CI	Age difference	95 % CI	Age difference	95 % CI
−20 %	−4.2	−6.3, −2.1	−3.6	−5.7, −1.5			−2.4	−4.5, −0.3	−1.8	−3.9, 0.3
−10 %	−3.6	−5.7, −1.5	−3.3	−5.4, −1.2			−2.7	−4.8, −0.6	−2.4	−4.5, −0.3
−5 %	−3.3	−5.4, −1.2	−3.15	−5.25, −1.05			−2,85	−4.95, −0.75	−2.7	−4.8, −0.6
0%					−3.0	−5.1, −0.9				
+5 %	−2.7	−4.8, −0.6	−2,85	−4.95, −0.75			−3.15	−5.25, −1.05	−3.3	−5.4, −1.2
+10 %	−2.4	−4.5, −0.3	−2.7	−4.8, −0.6			−3.3	−5.4, −1.2	−3.6	−5.7, −1.5
+20 %	−1.8	−3.9, 0.3	−2.4	−4.5, −0.3			−3.6	−5.7, −1.5	−4.2	−6.3, −2.1

Abbreviations: CI, confidence interval

[a]The parameter *a* is the difference in prevalence of U between unexposed and exposed girls [b]The parameter *b* is the mean monthly change/effect of U on age at attaining female axillary hair growth [c]All models adjusted for the same covariates as our main analyses [d]Age difference refers to the bias corrected effect estimates (mean monthly difference) and their 95 % CI confidence intervals.
